# Impact of Mobile Health Devices for the Detection of Atrial Fibrillation: Systematic Review

**DOI:** 10.2196/26161

**Published:** 2021-04-28

**Authors:** Tom E Biersteker, Martin J Schalij, Roderick W Treskes

**Affiliations:** 1 Leiden University Medical Center Leiden Netherlands

**Keywords:** eHealth, mHealth, telemedicine, cardiology, atrial fibrillation, systematic review

## Abstract

**Background:**

Atrial fibrillation (AF) is the most common arrhythmia, and its prevalence is increasing. Early diagnosis is important to reduce the risk of stroke. Mobile health (mHealth) devices, such as single-lead electrocardiogram (ECG) devices, have been introduced to the worldwide consumer market over the past decade. Recent studies have assessed the usability of these devices for detection of AF, but it remains unclear if the use of mHealth devices leads to a higher AF detection rate.

**Objective:**

The goal of the research was to conduct a systematic review of the diagnostic detection rate of AF by mHealth devices compared with traditional outpatient follow-up. Study participants were aged 16 years or older and had an increased risk for an arrhythmia and an indication for ECG follow-up—for instance, after catheter ablation or presentation to the emergency department with palpitations or (near) syncope. The intervention was the use of an mHealth device, defined as a novel device for the diagnosis of rhythm disturbances, either a handheld electronic device or a patch-like device worn on the patient’s chest. Control was standard (traditional) outpatient care, defined as follow-up via general practitioner or regular outpatient clinic visits with a standard 12-lead ECG or Holter monitoring. The main outcome measures were the odds ratio (OR) of AF detection rates.

**Methods:**

Two reviewers screened the search results, extracted data, and performed a risk of bias assessment. A heterogeneity analysis was performed, forest plot made to summarize the results of the individual studies, and albatross plot made to allow the *P* values to be interpreted in the context of the study sample size.

**Results:**

A total of 3384 articles were identified after a database search, and 14 studies with a 4617 study participants were selected. All studies but one showed a higher AF detection rate in the mHealth group compared with the control group (OR 1.00-35.71), with all RCTs showing statistically significant increases of AF detection (OR 1.54-19.16). Statistical heterogeneity between studies was considerable, with a Q of 34.1 and an *I*^2^ of 61.9, and therefore it was decided to not pool the results into a meta-analysis.

**Conclusions:**

Although the results of 13 of 14 studies support the effectiveness of mHealth interventions compared with standard care, study results could not be pooled due to considerable clinical and statistical heterogeneity. However, smartphone-connectable ECG devices provide patients with the ability to document a rhythm disturbance more easily than with standard care, which may increase empowerment and engagement with regard to their illness. Clinicians must beware of overdiagnosis of AF, as it is not yet clear when an mHealth-detected episode of AF must be deemed significant.

## Introduction

Atrial fibrillation (AF) is the most commonly diagnosed arrhythmia [1]. It may be paroxysmal (present for 30 seconds to 7 days), persistent (present for more than 7 days), or permanent [2]. Risk factors for AF are diverse and include advanced age, male gender, diabetes mellitus, hypertension, obesity, valvular disease, obstructive sleep apnea, heart failure, and previous myocardial infarction [3]. Among other symptoms, AF can cause palpitations, dyspnea, and tiredness. Patients can, however, be asymptomatic [4].

The worldwide prevalence of AF is increasing. This increase has been attributed to an aging population and increased prevalence of cardiovascular risk factors [5]. A European study has shown that the number of patients with diagnosed AF is expected to increase from a prevalence of 2.3% in 2010 to 3.5% to 4.3% in 2050 [6]. Due to an increased risk of stroke, AF is associated with increased risk of mortality [7]. Compared with patients with sinus rhythm, those with AF are found to have a 2.4-fold risk of stroke, and the risk of ischemic heart disease and development of chronic kidney disease are both increased 1.6-fold [8].

Early diagnosis of AF and prophylactic treatment for ischemic stroke with oral anticoagulants is therefore important, whether the AF is paroxysmal, persistent, or permanent and symptomatic or silent [2]. Moreover, it has been demonstrated that excessive supraventricular ectopic activity, defined as the presence of either ≥30 premature atrial contractions (PACs) per hour daily or any runs of ≥20 PACs, increases the risk of stroke in patients with a CHA_2_DS_2_-VASc (congestive heart failure, hypertension, age ≥75 years, diabetes mellitus, stroke or transient ischemic attack [TIA], vascular disease, age 65 to 74 years, sex category) score of ≥2 by 2.4% [9].

Traditionally, patients are diagnosed with AF using a 12-lead electrocardiogram (ECG). In case of suspected paroxysmal AF, it is possible to perform prolonged monitoring via Holter registration. However, as paroxysmal AF is often silent and patients can have vast periods of sinus rhythm, diagnosing paroxysmal AF is a challenge [10].

Over the last decade, consumer grade health monitoring devices have been developed and marketed as beneficial for personal health monitoring [11]. Among those devices are several different smartphone connectable ECG devices. The majority are lead-I ECG devices, handheld instruments that register lead I of the ECG, measuring the electric current generated by the myocardium by using the fingers of the right and the left hand [12]. These devices are typically used for spot-checks. Another group of devices is meant for continuous monitoring and involve patches that stick to the chest and allow monitoring of the heart rate and rhythm continuously for up to 2 weeks [13]. Both groups of devices can be seen as mobile health (mHealth) devices and used for AF screening [12].

Studies have been done to assess the accuracy of mHealth devices compared with 12-lead ECGs. A recent systematic review suggests several mHealth devices are suitable in the use of detecting AF, based on the sensitivity and specificity of these devices [14]. However, it is still unclear if and to what extent the use of mHealth devices leads to higher detection rates of AF. Therefore, the objective of this systematic review is to evaluate studies comparing the detection rate of AF by mHealth devices with more traditional outpatient follow-up.

## Methods

### Literature Review and Definitions

A systematic literature review was conducted to evaluate the efficacy of mHealth devices using standard (traditional) care as the reference standard in people with an indication for follow-up for a suspected arrythmia (eg, after catheter ablation or electrical cardioversion) or in cases of an acute emergency department presentation with (near) syncope or palpitations where no arrhythmia could be found at the time of presentation. The efficacy of mHealth was defined as the detection rate of AF by a smartphone-connectable ECG device, either a handheld electronic device or patch-like device attached to the study subject’s chest or by requiring subject to send an ECG transtelephonically. Standard care was defined as follow-up via a general practitioner or regular outpatient clinic visit with a standard 12-lead ECG or Holter monitoring. This systematic review was conducted and reported by following the Cochrane Handbook for Systematic Reviews of Interventions [15].

### Eligibility Criteria

The eligibility criteria for studies to be included in this systematic review were as follows:

- Published studies comparing mHealth devices with standard care in patients with an indication for follow-up via ECG or Holter monitoring
- Studies with AF detection as a primary or secondary outcome measure
- Studies conducted in people aged 16 years and older reporting demographic data such as patient characteristics, study setting, sample size, and data points
- Studies performed in a clinical or outpatient setting
- Studies in patients without an internal cardioverter defibrillator, pacemaker, or ventricular assist device

Studies had to be published in English or Dutch to be selected. If a study has been indexed in multiple databases, only the PubMed version was included.

### Literature Search Strategy

The search strategy is presented in Multimedia Appendix 1. No study design filters were applied, and all electronic databases were searched for articles from Jan 1, 2005, until February 19, 2020. The following databases were searched: Medline, Embase, PubMed, Web of Science, Emcare, Academic Search Premier, and the Cochrane Library. The search results were managed using EndNote X9 software (Clarivate Analytics). Relevant studies and reviews were manually searched to identify other possible relevant studies.

### Article Selection and Data Synthesis

A 2-stage process was used for inclusion in the review. Two reviewers (TB, RT) first independently screened all titles and abstracts of the identified studies to find potentially relevant studies. The same reviewers then assessed the full-text articles independently for the eligibility criteria. Any disagreements were resolved by consensus.

### Risk of Bias Assessment

Risk of bias was assessed with the RoB 2 (Risk of Bias 2) tool for randomized controlled trials (RCTs) and the ROBINS-I (Risk of Bias in Nonrandomized Studies of Interventions) tool for nonrandomized studies [16,17]. This is in accordance with the Cochrane Handbook’s recommendations [15]. The risk of bias had 3 levels: low risk of bias, some concerns, and high risk of bias.

### Summary Measures

The primary outcome measure of this systematic review was the odds ratio (OR) of AF detection, comparing mHealth devices to standard care. The PATCH-ED (Patch Monitor in Patients With Unexplained Syncope After Initial Evaluation in the Emergency Department) and IPED (Investigation of Palpitations in the Emergency Department) study groups reported no events in the control groups [18,19]. Therefore, the Haldane correction was used [20]. A heterogeneity analysis between studies was performed with a chi-square test [15]. A forest plot was made to summarize the results of individual studies. Finally, an albatross plot was made to allow the *P* values to be interpreted in the context of study sample size. The contour lines of albatross plots are formed by hypothetical effect sizes [21]. In this case, this concerns odds ratios due to the outcome being dichotomous. The forest and albatross plots were made in Matlab (The Mathworks Inc).

## Results

### Study Selection

**Figure 1:**
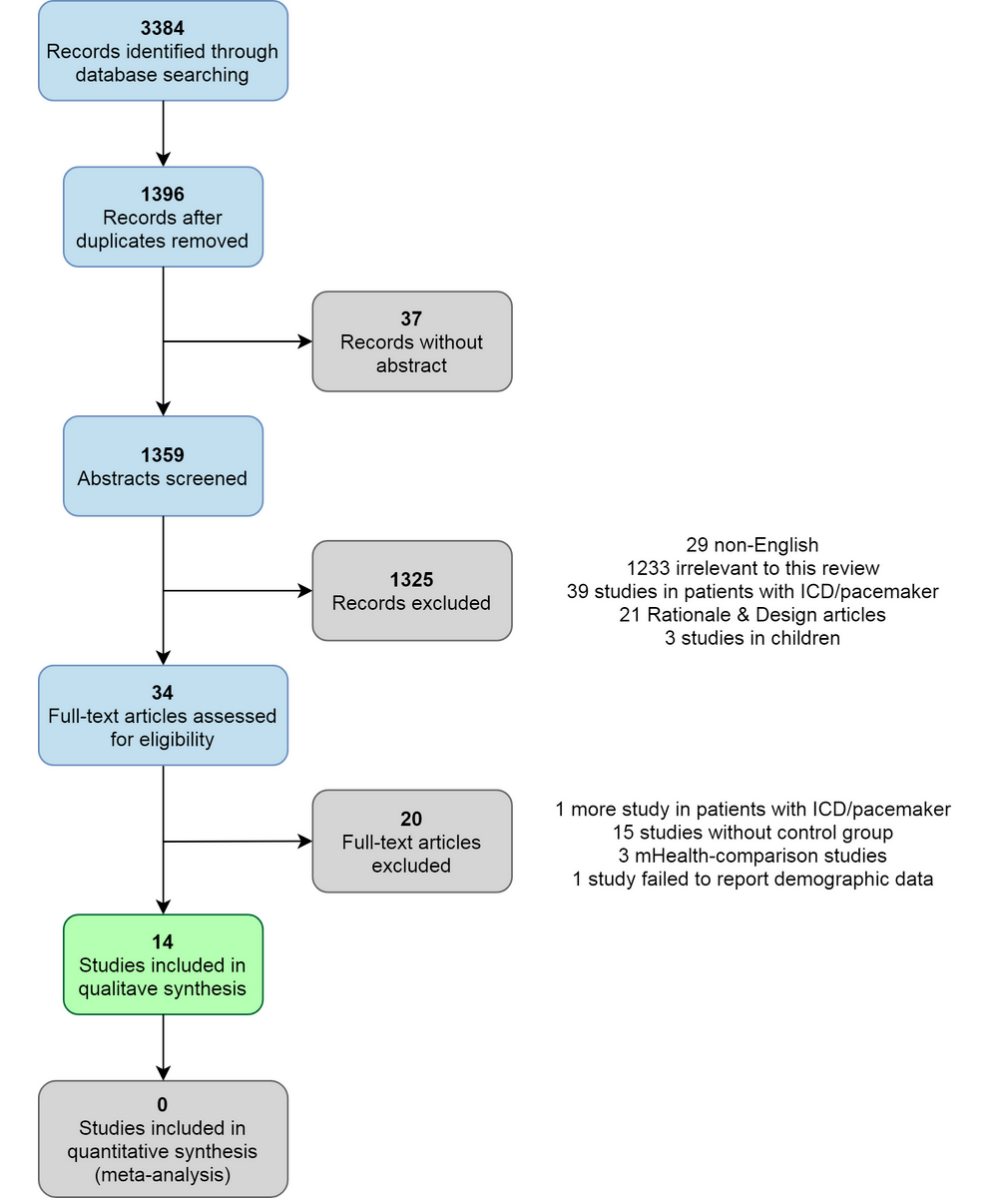
Study search and selection process.

As of October 19, 2020, a total of 3384 articles were obtained from the database searches. Two investigators (TB and RT) excluded 3350 studies based on the title and abstract. A total of 34 abstracts meeting the eligibility criteria were identified. After reviewing the full text, the reviewers chose 14 studies with a total of 4617 study subjects. The selection process is shown in Figure 1. The kappa statistic for interrater reliability was .81, showing substantial agreement between the 2 investigators [22].

### Study Characteristics

The 14 selected studies consist of 8 cohort studies, 4 RCTs, and 2 case-control studies [18,19,23-34]. Table 1 shows participant and study characteristics. Study populations were heterogenous: some studies included only patients without any history of AF, others included only patients with earlier documented AF. Participant genders varied between the study populations: 42% to 87% were male. Mean age varied from 44 to 73 years.

**Table 1:** Study characteristics.

Author, year, country	Study type	Patient charactertistics	Sample size; drop out; mean age; male	Intervention	Control	Follow-up	Primary outcome
Liu et al (2010), China [23]	Prospective cross-sectional	Catheter ablation patients	92; 0 (0%); 54 y^a^; 78% male	Transtelephonic ECG^b^ once daily	24 h Holter+ at complaints	90 d^c^	AF^d^ detection
Rosenberg et al (2013), US [24]	Prospective cross-sectional	Patients who are managed for AF, no definition was given	74; 0 (0%); 65 y; 55% male	ZioPatch	24 h Holter	14 d	AF detection
Barrett et al (2013), US [25]	Prospective cross-sectional	Outpatients with indication for Holter monitoring	146; 4 (2.7%); n/a^e^; n/a	ZioPatch	24 h Holter	14 d	Arrhythmia detection
Hendrikx et al (2014), Sweden [26]	Prospective cross-sectional	Patients with unexplained palpitations or presyncope	95; 0 (0%); 54 y; 44% male	Zenicor twice daily + 24 h^f^ Holter	24 h Holter	28 d	Arrhythmia detection
Kimura et al (2016), Japan [27]	Prospective cross-sectional	Catheter ablation patients	28; 2 (6.7%); 59 y; 87% male	CardioPhone twice daily	Monthly 24 h Holter	6 mo^g^	AF detection
Busch et al (2017), Germany [28]	Retrospective cross-sectional	Volunteers to join in an mHealth^h^ study	1678; n/a; 51 y; 48% male	SensorMobile twice daily	Single 12-lead ECG	28 d	AF detection
Halcox et al (2017), UK [29]	Single center, open label RCT^i^	≥65 y patients without AF at a GP^j^ practice	1001; 5 (0.5%); 73 y; 47% male	AliveCor Kardia twice a week	Follow-up at the GP	1 y	Time to diagnosis of AF
Hickey et al (2017), US [30]	Prospective, matched cohort study	Patients with a history of AF	46; 0 (0%); 55 y; 65% male	AliveCor Kardia once daily	Standard care (no added care)	6 mo	Atrial arrhythmia detection
Narasimha et al (2018), US [31]	Prospective cross-sectional	Patients with unexplained palpitations who underwent previous Holter monitoring	33; 5 (13.2%); 48 y; 42% male	AliveCor Kardia at complaints	External loop recorder	30 d	Arrhythmia detection
Reed et al (2018), Scotland [18]	Prospective, unmatched case-control study	≥16 y ER patients with unexplained syncope	689; 0 (0%); 67 y; 47% male	ZioPatch	Standard care (no added care)	14 d	Symptomatic rhythm detection
Reed et al (2019), Scotland [19]	Multicenter, open label RCT	≥16 y ER patients with unexplained palpitations or (pre)syncope	240; 2 (0.8%); 40 y; 44% male	Alivecor Kardia at complaints	Standard care (no added care)	90 d	Symptomatic rhythm detection
Goldenthal et al (2019), US [32]	Single center, open label RCT	Patients with documented AF, undergoing ablation or ECV^k^	238; 5 (2.1%); 61 y; 76% male	AliveCor Kardia daily and at complaints	Standard care (no added care)	6 mo	AF detection
Karunadas et al (2019), India [33]	Prospective cross-sectional	Admitted patients to cardiology ward who required monitoring	141; 0 (0%); 44 y; 53% male	WebCardio (patch)	24 h Holter	1 d	Arrhythmia detection
Kaura et al (2019), UK [34]	Multicenter, open label RCT	Non-AF patients with nonlacunar stroke or TIA^l^	116; 26 (22.4%); 70 y; 47% male	ZioPatch	24 h Holter	14 d	AF detection

^a^y: year.

^b^ECG: electrocardiogram.

^c^d: day.

^d^AF: atrial fibrillation.

^e^Not applicable.

^f^h: hour.

^g^mo: month.

^h^mHealth: mobile health.

^i^RCT: randomized controlled trial.

^j^GP: general practice.

^k^ECV: electrical cardioversion.

^l^TIA: transient ischemic attack.

A total of 9 studies used handheld devices such as the Kardia (AliveCor Inc) or Zenicor-ECG (Zenicor Medical Systems AB) as an intervention, while 5 studies used a patch such as the Zio (iRhythm Technologies Inc), which was placed on the participant’s chest [13,35,36]. The duration of the intervention was 1 to 14 days for studies with patches and 28 days to 1 year for studies with handheld devices. All studies published data about AF detection, although AF detection was the primary outcome in only 6 studies. A total of 4 studies used detection of any arrhythmia (AF, atrial flutter, supraventricular or ventricular tachycardia, sinus pauses of more than 3 seconds, and second- and third-degree atrioventricular blocks), and 2 other studies reported symptomatic arrhythmias as the primary outcome; 1 study used atrial arrhythmia detection and the final study reported the time to AF diagnosis as the primary outcome. One study reported a composite endpoint of AF, ventricular tachycardia, and sinus pauses of more than 3 seconds instead [25].

A total of 6 studies used 24-hour Holter monitoring as standard care, with 1 study adding another 24-hour Holter monitoring when study patients experienced an episode of palpitations and another study adding another 24-hour Holter monitoring every month, 6 times in total. However, 5 studies only saw patients back in the outpatient clinic or general practitioner. One study used an external loop recorder as standard care, activated at complaints during the entire follow-up duration, and the final study documented one extra standard ECG as standard care. Holter timing was at the start of the study in 4 of 6 studies that used Holter monitoring. In the other 2 studies, the timing of the Holter monitoring was unclear.

### Study Results

**Figure 2:**
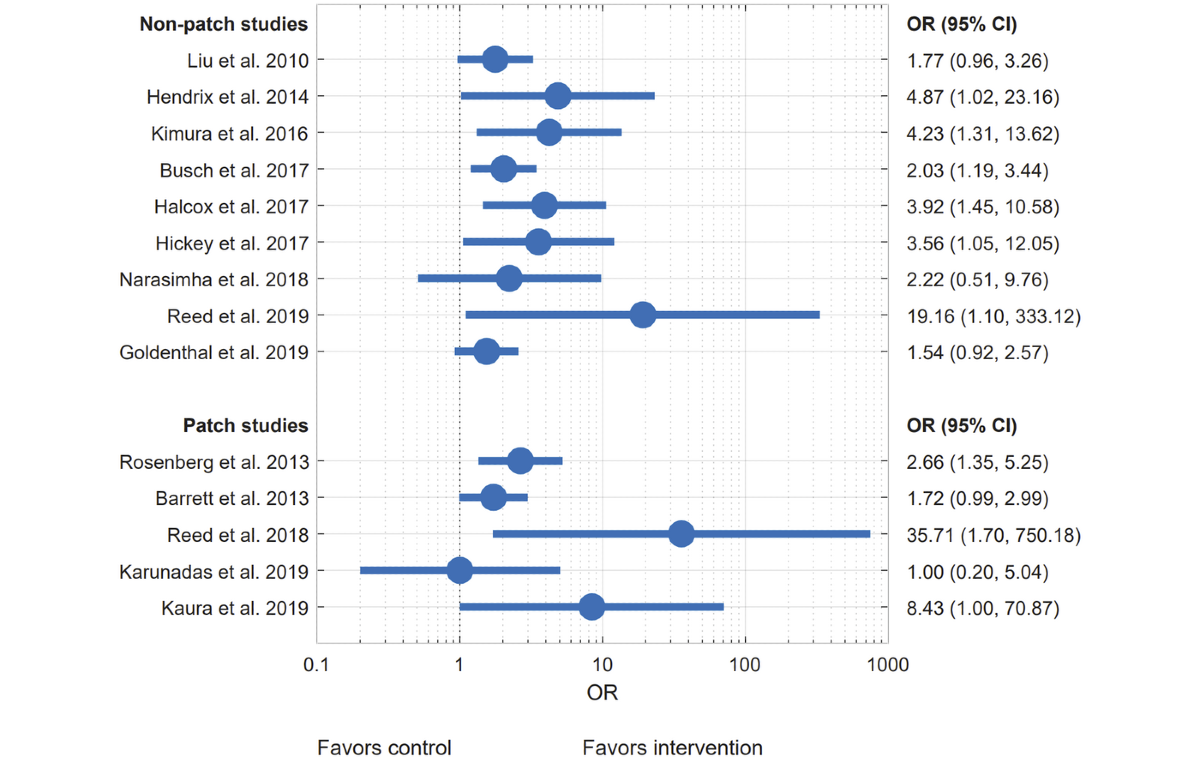
Forest plot of the study results. No pooling due to heterogeneity.

**Figure 3:**
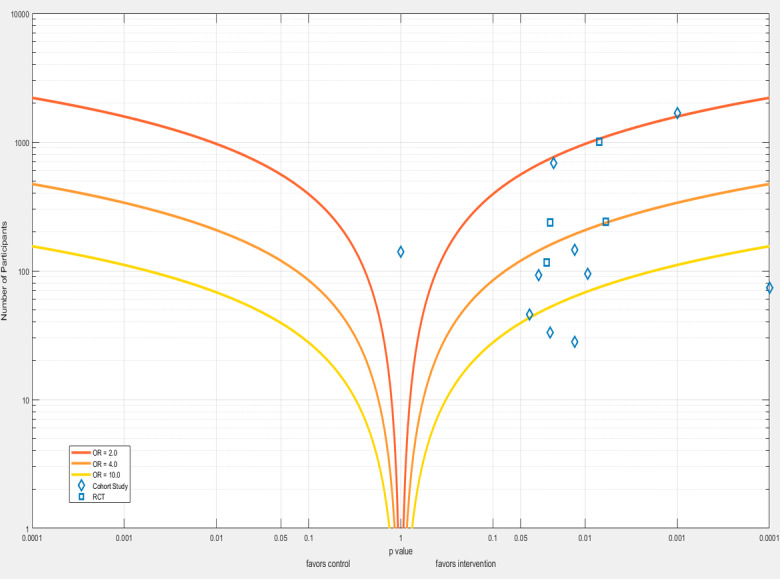
Albatross plot, with plotted odds ratio lines.

Table 2 shows the number of events throughout the studies. The individual study results are shown in a forest plot (Figure 2) but not pooled due to the considerable clinical and statistical heterogeneity. To show the *P* values in the context of the study sample size, an albatross plot is presented (Figure 3).

**Table 2:** Study outcomes.

Author		Sample size, n	Intervention group, n	Control group, n	Events (intervention), n (%)	Events (control), n (%)	Odds ratio (95% CI)
**Nonpatch studies**							
	Liu et al, 2010 [23]	92	—^a^	—	39 (42.4)	27 (29.2)	1.77 (0.96-3.26)
	Hendrikx et al, 2014 [26]	95	—	—	9 (9.5)	2 (2.1)	4.87 (1.02-23.16)
	Kimura et al, 2016 [27]	28	—	—	15 (53.6)	6 (21.4)	4.23 (1.31-13.62)
	Busch et al, 2017 [28]	1678	—	—	42 (2.6)	21 (1.3)	2.03 (1.19-3.44)
	Halcox et al, 2017 [29]	1001	500	501	19 (3.8)	5 (1.0)	3.92 (1.45-10.58)
	Hickey et al, 2017 [30]	46	23	23	14 (60.9)	7 (30.4)	3.56 (1.05-12.05)
	Narasimha et al, 2018 [31]	33	—	—	6 (18.2)	3 (9.1)	2.22 (0.51-9.76)
	Reed et al, 2019 [19]	240	124	116	9 (7.3)	0 (0)	19.16^b^ (1.10-333.12)
	Goldenthal et al, 2019 [32]	238	115	123	58 (50.4)	49 (41.5)	1.54 (0.92-2.57)
**Patch studies**							
	Rosenberg et al, 2013 [24]	74	—	—	38 (51.3)	21 (28.4)	2.66 (1.35-5.26)
	Barrett et al, 2013 [25]	146	—	—	41 (28.1)	27 (18.5)	1.72 (0.99-2.99)
	Reed et al, 2018 [18]	689	86	603	2 (2.3)	0 (0)	35.71^b^ (1.70-750.18)
	Karunadas et al, 2019 [33]	141	—	—	3 (2.1)	3 (2.1)	1.00 (0.20-5.04)
	Kaura et al, 2019 [34]	116	56	60	7 (16.3)	1 (2.1)	8.43 (1.00-70.87)

^a^Not applicable.

^b^Haldane correction applied.

All studies showed a higher AF detection rate in the mHealth group compared with the control group except the study by Karunadas, which showed an equal number of events (3; 2.1%) in both groups [33]. This study used an mHealth patch for 1 day and compared it to Holter monitoring performed on the same day. The 24-hour to 72-hour patch data have been disregarded for the analysis.

All RCTs showed a statistically significant improvement of AF detection with mHealth devices. ORs were 3.92 (95% CI 1.45-10.58) for the REHEARSE-AF (Assessment of Remote Heart Rhythm Sampling Using the AliveCor Heart Monitor to Screen for Atrial Fibrillation) trial, 19.16 (95% CI 1.10-333.12) for IPED, 1.54 (95% CI 0.92-2.57) in the iHeart (Information Technology Approach to Implementing Depression Treatment in Cardiac Patients) trial, and 8.43 (95% CI 1.00-70.87) in the EPACS (Early Prolonged Ambulatory Cardiac Monitoring in Stroke) trial.

### Statistical Heterogeneity

The 14 selected studies showed a variety of populations, interventions, and outcomes and are therefore considerably clinically heterogenic. A chi-square test was conducted to assess statistical heterogeneity, which showed a Q of 34.1 and an *I*^2^ of 61.9, and therefore the studies show considerable statistical heterogeneity.

### Quality Appraisal

**Figure 4:**
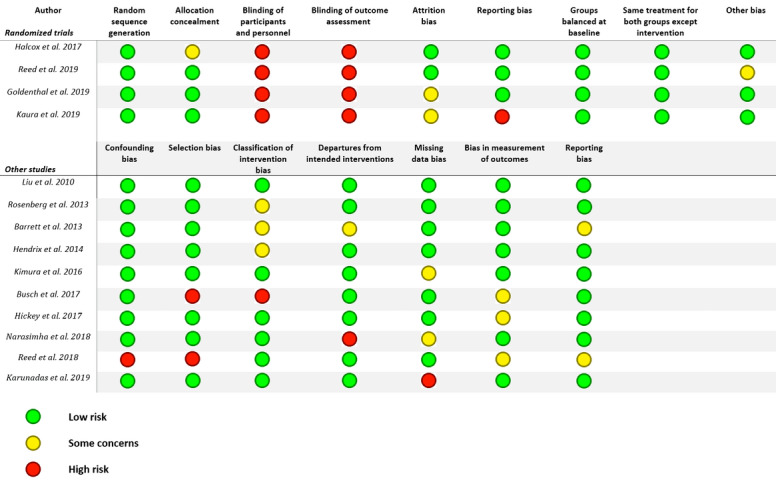
Risk of bias assessment. Randomized trials were assessed with the ROB 2 (Risk of Bias 2) tool, while ROBINS-I was used for nonrandomized studies. ROBINS-I: Risk of Bias in Nonrandomized Studies of Interventions.

Figure 4 presents the generic risk of bias, assessed with the RoB 2 and ROBINS-I tools. In the selected RCTs, blinding of participants was not possible due to the nature of the intervention. Of all selected RCTs, one had a high risk of bias on the outcome data. Kaura et al [34] reported a dropout of 22.4% and did not address this data in the report. This was also true for the RCT by Goldenthal et al [32], but the dropout in this trial was just 2.1%. As for allocation concealment in the trial carried out by Halcox et al [29], no clarity was provided in the method section of the paper.

Of the nonrandomized studies, the studies by Liu et al [23], Rosenberg et al [24], Hendrikx et al [26], Kimura et al [27], and Hickey et al [30] were scored as strong. Several studies showed an intermediate risk of bias. Barrett et al [18] reported no baseline characteristics, and Holter timing was unclear. Narasimha et al [25] reported a dropout of 13.2% but performed separate per-protocol and intention-to-treat analyses. Reed et al [31] used unmatched cohorts with several parameters not being known or stated. Also, there was a time interval of 7 to 8 years between gathering of the data in the intervention and control cohorts.

Two studies showed a high risk of bias. Busch et al [28] used data from a registry, in which the study subjects were volunteers willing to participate in an mHealth study. Karunadas et al [33] reported no baseline characteristics, and only WebCardio data from the first 24 hours were used. The 24-hour to 72-hour data, although gathered, were not reported.

## Discussion

### Summary of Evidence

The main finding of this systematic review of 14 studies is the increased AF detection rate when using mHealth devices compared with standard follow-up. Moreover, the 4 RCTs included all showed a statistically significant difference. However, there was a considerable clinical and statistical interstudy heterogeneity. The results of all studies but one show that mHealth devices lead to an increased detection of AF.

An argument can be made that conducting more (spot) measurements will automatically lead to more diagnoses of any illness. However, as AF is often only present for a short period of time and untraceable once sinus rhythm is restored, the clinical implications of the opportunity for conducting more spot measurements could be of importance with regard to stroke risk, for example. Following standard care does not allow patients to record their ECG without a delay, as they must visit their care provider or call an ambulance. Meanwhile, a paroxysm of AF may already have disappeared. Smartphone-connectable ECG devices could therefore provide patients with the opportunity to act immediately by documenting their rhythm disturbance. This is not only true for AF but also for other paroxysmal arrhythmias.

Although both handheld devices and patches lead to an increased AF detection rate, there may be a different use case to both groups of devices. Patches could be seen as prolonged Holter monitoring. The Zio patch can remain on the body for up to 14 days [13]. Handheld devices are used to do spot measurements for a longer period of time and can therefore only be used for screening or in patients with complaints that could fit with a rhythm disturbance. Therefore, the benefit of patches over handheld devices is that asymptomatic rhythm disturbances may be diagnosed with the use of a patch, although patient-triggered recordings with handheld ECG devices may be a more viable solution when a longer period of follow-up is indicated.

### Potential of mHealth for Population-Based Screening

Smartphone-connectable ECG devices cannot only be used in patients with a suspected paroxysmal rhythm disturbance but also for screening purposes. As stroke has been found to be the first symptom of AF in 37% of patients aged younger than 75 years with no history of cardiovascular diseases, secondary prevention in the form of screening risk groups for AF de novo may be of clinical relevance [37]. When it comes to screening for AF, there are several possibilities. Individuals can be screened regardless of medical history (systematic screening), on presenting to a physician for issues unrelated to AF (systematic opportunistic screening), or based on the presence of AF-associated risk factors (targeted screening). A recent meta-analysis has shown opportunistic screening, with a number needed to screen of 170, to be a likely cost-effective use of resources [38]. However, the number needed to screen varies between age groups and is found to be lowest, 83, in patients aged older than 65 years, against 926 for ages 60 to 64 years and 1089 for patients aged younger than 60 years, and therefore screening might be most opportune in people aged older than 65 years [39]. A very recent study using a Monte Carlo simulation to assess the cost-effectiveness of screening for AF with mHealth devices using 30,000 patients per CHA_2_DS_2_-VASc score (1-9) has found this type of screening to cause increased health care costs but a reduction in the incidence of stroke [40]. Several mHealth studies have used a systematic opportunistic screening approach such as screening for AF with handheld devices in individuals who visit pharmacies or those who visit their general practitioner for a flu vaccination [41-45]. These studies have all concluded handheld smartphone-connectable ECG devices to be viable screening tools.

### Clinical Implications

In this era of mHealth, patients are increasingly able to take (spot) measurements by using smartphone-connectable ECG devices, as those devices are commercially available. However, no consensus exists within the scientific community whether each episode of AF should be seen as clinically significant. AF is traditionally defined as an irregular arrhythmia without visible P waves lasting 30 seconds or more or documented on a standard 10-second 12-lead ECG [46]. The Kardia and other devices that register a lead-I ECG document a period of 30 seconds [35]. However, the clinical significance of a short paroxysm of AF is debated. Looking at AF ablation patients, it is known that the quality of life response is proportional to the burden rather than to a short-lived event and the AF burden is also a better predictor for stroke risk compared solely with a history of AF [47,48]. A recent study in patients with pacemakers tested various AF episode duration thresholds and found that patients with initial AF events up to 3.8 hours only had a median AF burden of 0.2% compared with 9.5% for those with initial AF episodes of more than 3.8 hours. This was a statistically significant difference with a *P* value of <.0001 [49].

### Limitations

Due to considerable clinical and statistic heterogeneity, with an *I*^2^ of 61.9, the results of the included studies could not be pooled into a meta-analysis. The study populations varied from healthy adults to patients with an extensive history of AF, interventions ranged from short-term follow-up with a patch to long-term follow-up with a handheld device, and primary outcomes were also diverse. These differences led to a wide spread in the number of detected cases of AF, from 1% to 3% in the study by Busch et al [28] to 30% to 61% in the study by Hickey et al [30]. Instead of performing a meta-analysis, a forest plot without a diamond and an albatross plot were made. Furthermore, participants in RCTs could not be blinded due to the nature of the intervention. This is a small problem, however, since a diagnosis of AF is not a subjective end point.

### Conclusion

This systematic review reflects on 14 studies with different populations, interventions, and (primary) outcomes. A total of 13 studies found an increased number of AF diagnoses with the use of an mHealth intervention compared with standard care, with the remaining study by Karunadas et al [33] showing equal effectiveness. All 4 RCTs showed a statistically significant result in favor of the mHealth intervention. Due to considerable clinical and statistical heterogeneity, individual study results could not be pooled into a meta-analysis, and as a result, it cannot be concluded that those mHealth interventions are effective in certain populations or every population. However, smartphone-connectable ECG devices provide patients with the ability to document a rhythm disturbance more easily than with standard care, and with the introduction of more mHealth devices and specifically devices that can diagnose AF like the Apple Watch (Apple Inc) and Move ECG (Withings) [50,51], this is unlikely to change. With increased patient expectations and the increased empowerment and engagement with regard to their illness that mHealth devices may provide [52], future patients may request mHealth to be a part of their standard follow-up. However, as it is not yet clear when an mHealth-detected episode of AF should be deemed significant [48], clinicians must beware of overdiagnosis of AF and, sequentially, overtreatment with oral anticoagulants.

## Appendix

Multimedia Appendix 1 Search strategy.

## Abbreviations

AF: atrial fibrillation
CHA_2_DS_2_-VASc: congestive heart failure, hypertension, age ≥75 years, diabetes mellitus, stroke or transient ischemic attack (TIA), vascular disease, age 65 to 74 years, sex category
ECG: electrocardiogram
EPACS: Early Prolonged Ambulatory Cardiac Monitoring in Stroke
iHeart: An Information Technology Approach to Implementing Depression Treatment in Cardiac Patients
IPED: Investigation of Palpitations in the Emergency Department
mHealth: mobile health
OR: odds ratio
PAC: premature atrial contraction
PATCH-ED: Patch Monitor in Patients With Unexplained Syncope After Initial Evaluation in the Emergency Department
RCT: randomized controlled trial
REHEARSE-AF: Assessment of Remote Heart Rhythm Sampling Using the AliveCor Heart Monitor to Screen for Atrial Fibrillation
RoB 2: Risk of Bias 2
ROBINS-I: Risk Of Bias in Nonrandomized Studies of Interventions
