# Supplementary material for: Impact of Mobile Health Devices for the Detection of Atrial Fibrillation: Systematic Review
Source: JMIR Mhealth Uhealth. 2021 Apr 28;9(4):e26161. doi: 10.2196/26161 (PMC8116993; doi:10.2196/26161)
Supplement: Multimedia Appendix 1 [file mhealth_v9i4e26161_app1.docx]

((("palpitation"[tw] OR "palpitations"[tw] OR "Arrhythmias, Cardiac"[mesh:noexp] OR "Atrial Flutter"[mesh] OR "Tachycardia"[mesh] OR "Ventricular Fibrillation"[mesh] OR "Ventricular Flutter"[mesh] OR "near collapse"[tw] OR "collapse"[tw] OR "Syncope"[mesh] OR "syncope"[tw] OR "Dizziness"[Mesh] OR "Dizziness"[tw] OR "light headedness"[tw] OR "lightheadedness"[tw] OR "Electric Countershock"[Mesh] OR "Electric Countershock"[tw] OR "cardioversion"[tw] OR "Ablation Techniques"[mesh:noexp] OR "Catheter Ablation"[mesh] OR "Cryosurgery"[mesh] OR "Ablation"[tw] OR "Catheter Ablation"[tw] OR "Cryosurgery"[tw] OR "Electrocardiography, Ambulatory"[Mesh] OR "Ambulatory Electrocardiography"[tw] OR "holter"[tw] OR "Photoplethysmography"[Mesh] OR "photoplethysmography"[tw] OR photoplethysmogr*[tw] OR "Atrial Fibrillation"[Mesh] OR "Atrial Fibrillation"[tw] OR Atrial Fibrill*[tw] OR "Auricular Fibrillation"[tw] OR Auricular Fibrillat*[tw] OR "Atrial Flutter"[Mesh] OR "Atrial Flutter"[tw] OR Atrial Flutter*[tw] OR "Auricular Flutter"[tw] OR Auricular Flutter*[tw]) AND ("AliveCor"[ti] OR "Kardia"[ti] OR "Zenicor"[ti] OR "CardioPhone"[ti] OR "ZioPatch"[ti] OR "Zio"[ti] OR "mydiagnostick"[ti] OR "iheart"[ti] OR "mhealth"[ti] OR "m-health"[ti] OR "mobile health"[ti] OR "wearable technology"[ti] OR "wearable technologies"[ti] OR "Smartphone"[ti] OR "Smartphones"[ti] OR iphon*[ti] OR "ipad"[ti] OR ipad*[ti] OR "mobile phones"[ti] OR "mobile phone"[ti] OR "mobile apps"[ti] OR "mobile apps"[ti] OR "app"[ti] OR "apps"[ti] OR webapp*[ti] OR "ehealth"[ti] OR "e-health"[ti] OR "smart technology"[ti] OR smart technol*[ti] OR "Telemedicine"[majr:noexp] OR "Telerehabilitation"[majr] OR "Telemedicine"[ti] OR "Telerehabilitation"[ti] OR "telehealth"[ti] OR "ihealth"[ti] OR "i-health"[ti] OR e-consult*[ti] OR econsult*[ti])) OR (("palpitation"[ti] OR "palpitations"[ti] OR "Arrhythmias, Cardiac"[majr:noexp] OR "Atrial Flutter"[majr] OR "Tachycardia"[majr] OR "Ventricular Fibrillation"[majr] OR "Ventricular Flutter"[majr] OR "near collapse"[ti] OR "collapse"[ti] OR "Syncope"[majr] OR "syncope"[ti] OR "Dizziness"[majr] OR "Dizziness"[ti] OR "light headedness"[ti] OR "lightheadedness"[ti] OR "Electric Countershock"[majr] OR "Electric Countershock"[ti] OR "cardioversion"[ti] OR "Ablation Techniques"[majr:noexp] OR "Catheter Ablation"[majr] OR "Cryosurgery"[majr] OR "Ablation"[ti] OR "Catheter Ablation"[ti] OR "Cryosurgery"[ti] OR "Electrocardiography, Ambulatory"[majr] OR "Ambulatory Electrocardiography"[ti] OR "holter"[ti] OR "Photoplethysmography"[majr] OR "photoplethysmography"[ti] OR photoplethysmogr*[ti] OR "Atrial Fibrillation"[majr] OR "Atrial Fibrillation"[ti] OR Atrial Fibrill*[ti] OR "Auricular Fibrillation"[ti] OR Auricular Fibrillat*[ti] OR "Atrial Flutter"[majr] OR "Atrial Flutter"[ti] OR Atrial Flutter*[ti] OR "Auricular Flutter"[ti] OR Auricular Flutter*[ti]) AND ("AliveCor"[tw] OR "Kardia"[tw] OR "Zenicor"[tw] OR "CardioPhone"[tw] OR "ZioPatch"[tw] OR "Zio"[tw] OR "mydiagnostick"[tw] OR "iheart"[tw] OR "mhealth"[tw] OR "m-health"[tw] OR "mobile health"[tw] OR "wearable technology"[tw] OR "wearable technologies"[tw] OR "Smartphone"[tw] OR "Smartphones"[tw] OR iphon*[tw] OR "ipad"[tw] OR ipad*[tw] OR "mobile phones"[tw] OR "mobile phone"[tw] OR "mobile apps"[tw] OR "mobile apps"[tw] OR "app"[tw] OR "apps"[tw] OR webapp*[tw] OR "ehealth"[tw] OR "e-health"[tw] OR "smart technology"[tw] OR smart technol*[tw] OR "Telemedicine"[mesh:noexp] OR "Telerehabilitation"[mesh] OR "Telemedicine"[tw] OR "Telerehabilitation"[tw] OR "telehealth"[tw] OR "ihealth"[tw] OR "i-health"[tw] OR e-consult*[tw] OR econsult*[tw])) OR (("palpitation"[ti] OR "palpitations"[ti] OR "Arrhythmias, Cardiac"[majr:noexp] OR "Atrial Flutter"[majr] OR "Tachycardia"[majr] OR "Ventricular Fibrillation"[majr] OR "Ventricular Flutter"[majr] OR "near collapse"[ti] OR "collapse"[ti] OR "Syncope"[majr] OR "syncope"[ti] OR "Dizziness"[majr] OR "Dizziness"[ti] OR "light headedness"[ti] OR "lightheadedness"[ti] OR "Electric Countershock"[majr] OR "Electric Countershock"[ti] OR "cardioversion"[ti] OR "Ablation Techniques"[majr:noexp] OR "Catheter Ablation"[majr] OR "Cryosurgery"[majr] OR "Ablation"[ti] OR "Catheter Ablation"[ti] OR "Cryosurgery"[ti] OR "Electrocardiography, Ambulatory"[majr] OR "Ambulatory Electrocardiography"[ti] OR "holter"[ti] OR "Photoplethysmography"[majr] OR "photoplethysmography"[ti] OR photoplethysmogr*[ti] OR "Atrial Fibrillation"[majr] OR "Atrial Fibrillation"[ti] OR Atrial Fibrill*[ti] OR "Auricular Fibrillation"[ti] OR Auricular Fibrillat*[ti] OR "Atrial Flutter"[majr] OR "Atrial Flutter"[ti] OR Atrial Flutter*[ti] OR "Auricular Flutter"[ti] OR Auricular Flutter*[ti]) AND ("remote communication"[tw] OR "remote computer"[tw] OR "remote computers"[tw] OR "remote consultation"[tw] OR "remote health care"[tw] OR "remote healthcare"[tw] OR "remote monitoring"[tw] OR "remote system"[tw] OR "remote systems"[tw] OR "remote technologies"[tw] OR "remote technology"[tw] OR "teleconsultation"[tw] OR teleconsult*[tw] OR "Text Messaging"[tw] OR text messag*[tw] OR "texting"[tw] OR "short message service"[tw] OR "Electronic Mail"[mesh] OR "Electronic Mail"[tw] OR e-mail*[tw] OR email*[tw] OR "SMS"[tw] OR mobile*[tw] OR "Reminder Systems"[mesh] OR "Reminder Systems"[tw] OR "Reminder System"[tw] OR "Reminder Device"[tw] OR "Reminder Devices"[tw] OR "reminder messages"[tw] OR "reminder message"[tw]) AND "clinical trial"[ptyp])) NOT ("Animals"[mesh] NOT "Humans"[mesh])
